# Bilateral basal ganglia infarction and bilateral thalamic lesions in sudanese pediatric patient with COVID‐19 and malaria co‐infection, a case report

**DOI:** 10.1002/ccr3.5322

**Published:** 2022-02-02

**Authors:** Yassin Abdelrahim Abdalla, Mohannad Abdalfdeel Almahie Shaban, Khabab Abbasher Hussien Mohamed Ahmed, Mazin S. Haroun, Moh. Mah. Fadelallah Eljack, Khalid Sidahmed Eltom, Rasha Sidahmed Elhassan

**Affiliations:** ^1^ Department of Internal Medicine Faculty of Medicine and Health Sciences Omdurman Islamic University Khartoum Sudan; ^2^ Faculty of Medicine Alzaiem Alazhary University Khartoum Sudan; ^3^ Faculty of Medicine University of Khartoum Khartoum Sudan; ^4^ Faculty of Medicine Medani Heart Centre University of Bakht Alruda Wad Medani Sudan; ^5^ Alribat teaching hospital Khartoum Sudan

**Keywords:** COVID‐19, encephalitis, malaria, neurology, pediatric, stroke

## Abstract

COVID‐19 is of uncommon diagnosis in pediatric with their presentation in much of time of a non‐specific entity; here, we experienced the case of a 2‐year‐old female with malaria presented with fever, cough, rhinorrhea, hemoptysis, and convulsion diagnosed as COVID‐19, complicated with encephalitis, received treatment, and improved over weeks.

## Graphical abstract

COVID‐19 infection in pediatrics is still not understood. Malaria, on the other hand, has its own consequences in pediatrics. Here, we report the case of a pediatric patient with COVID‐19 and malaria co‐infection and how did it was managed.

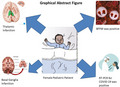

## 1 INTRODUCTION

Since 2019 when the first SARS‐CoV‐2 case was reported in China, different and variable clinical presentations with a wide variety of symptoms have been documented by literature ranging from common manifestations to unfamiliar presentations. From multiple reported cases in early 2020 in China, 2% of the victims were children.^1^ The pediatric population tends to have relatively mild and non‐specific symptoms compared to adults. Symptomatic infection is less common. In those with symptoms, fever and cough are the most common manifestations.^2^, ^3^, ^4^ Symptoms in children can be related to upper respiratory tract, pneumonia, or gastrointestinal symptoms. It can be classified into mild, moderate, and severe infections. Severe infection occurs less frequently and includes respiratory distress, decreased oxygen saturation, cyanosis, feeding difficulties, loss of consciousness, seizures, or shock.^12^ Cases may present with neurological symptoms related to either the peripheral nervous system or central nervous system. Literature reported paraparesis, headache, encephalopathy, and epileptic seizures as a sequel of COVID‐19.^2^, ^3^ In this report, we describe a child with COVID‐19 and malaria co‐infection who presented with convulsions and prolonged loss of consciousness.

## 2 CASE PRESENTATION

A 2‐year old previously well female presented with intermittent high‐grade fever, cough, and rhinorrhea for 1 day. The patient sought medical advice and her blood film for malaria (BFFM) came negative with high total white blood cells count (TWBC) and the patient received four doses of benzylpenicillin but with minimal improvement. One week later, the patient developed high‐grade fever and sought advice, again BFFM was done and this time came positive. She was put on injectable artemether and ceftriaxone and discharged home. One day later, the patient developed two attacks of generalized convulsion for 2 min and 3 min, respectively, with complete resolution between them and the patient was taken to ER where the third attack of convulsions occurred during assessment which was associated with hematemesis; then, the patient was unconscious after that.

On examination, the patient was vitally stable but deeply comatose; in decorticate position, with Glasgow Coma Scale (GCS) of 3. There was hypertonia with exaggeration of all reflexes in both the upper and lower limbs bilaterally, with bilateral up‐going plantar reflex, and her both pupils were in pinpoint shape but still reactive to light, with no other signs of other cranial nerves’ defect. The power could not be assessed. Cardiac examination was unremarkable. Her lung auscultation revealed diffuse crackles all over the chest, with normal air entry. The liver was palpable 4cm below the costal margin.

Investigations requested were unremarkable apart from leukopenia with lymphocytopenia; the result of RT‐PCR for COVID‐19 was positive (Table 1).

**TABLE 1:** Investigation result

Test	Result	Normal range
CBC		
TWBC	2.7 × 10^3	N.R 10–26
RBC	4.74 × 10^6	N.R 4.1–6.7
HGB	10.8 g/dl	
HCT	32.8%	N.R 44–50 low
MCV	69.3 fl	102–115 low
MCH	22.7 pg	33–39 low
MCHC	32.9 g/dl	
PLT	299 × 10^3/	150–450
LYM%	39	N.R %20–40
MXD %	14.6	1–15
NEUT%	46.4%	
LYM	1.1 × 10^3/ml	2.5–10.5
NEUT	1.3 × 10^3/ml	
MXD	0.3	
RDW‐CV	18.6	11–16
CRP	7	Up–10
Bleeding profile		
INR	0.8	
PT	13.9	
PT control	15	
Liver profile		
Total portion	6.5 g/dl	6.6–8.7
Serum albumin	3.9 g/dl	3.4–4,8
Bilirubin (total)	0.2 mg/dl	Less than 1.1
Bilirubin (direct)	0.1 mg/dl	Less than 0.3
Bilirubin (indirect)	0.1 mg/dl	
ALT (GPT)	27 U/L	Up–45
AST (GOP)	37 U/l	Up–42
ALP	232 U/L	Child 727
Urea and electrolytes		
Blood urea	55 mg/dl	10–50
Serum creatinine	0.5 mg/dl	F(o.6–1.1) M(0.7–1.4)
Na^+^	140 mmol/L	135–145
K^+^	4.5 mmol/L	3.5–5.0

**FIGURE 1:**
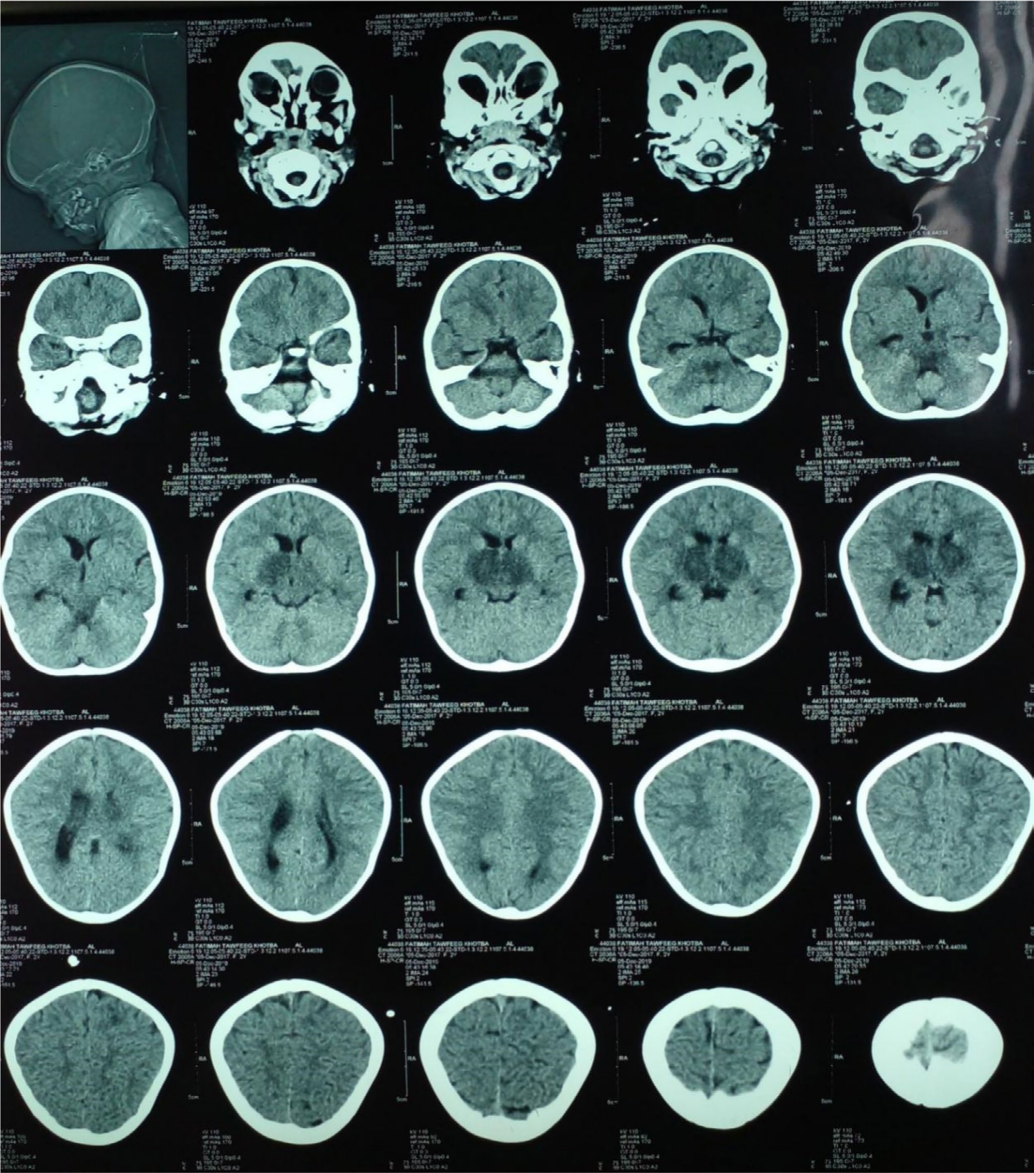
CT of the brain was done which revealed bilateral basal ganglia infarction

**FIGURE 2:**
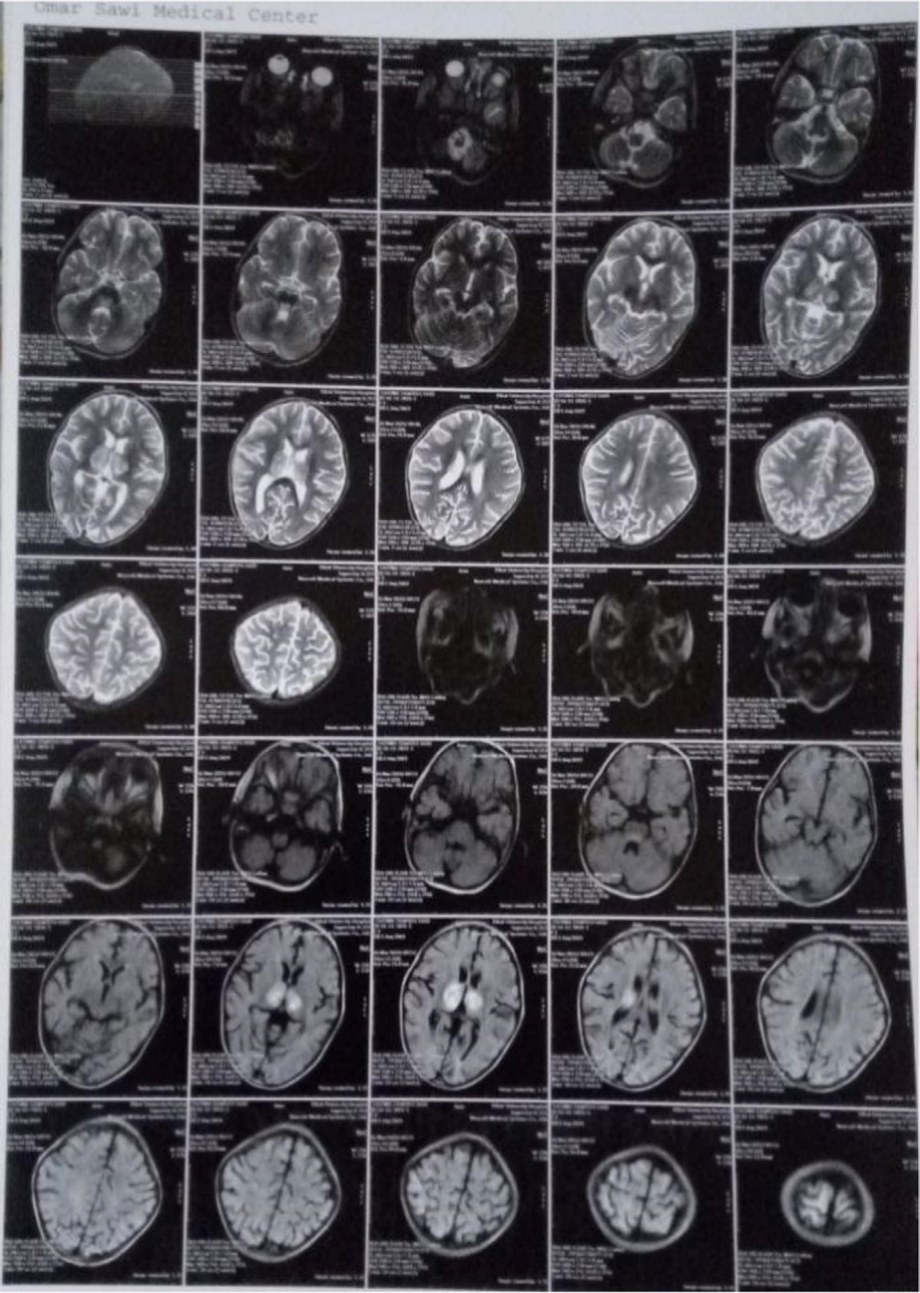
MRI reveals bilateral thalamic lesion due to viral encephalitis or vascular insult

**FIGURE 3:**
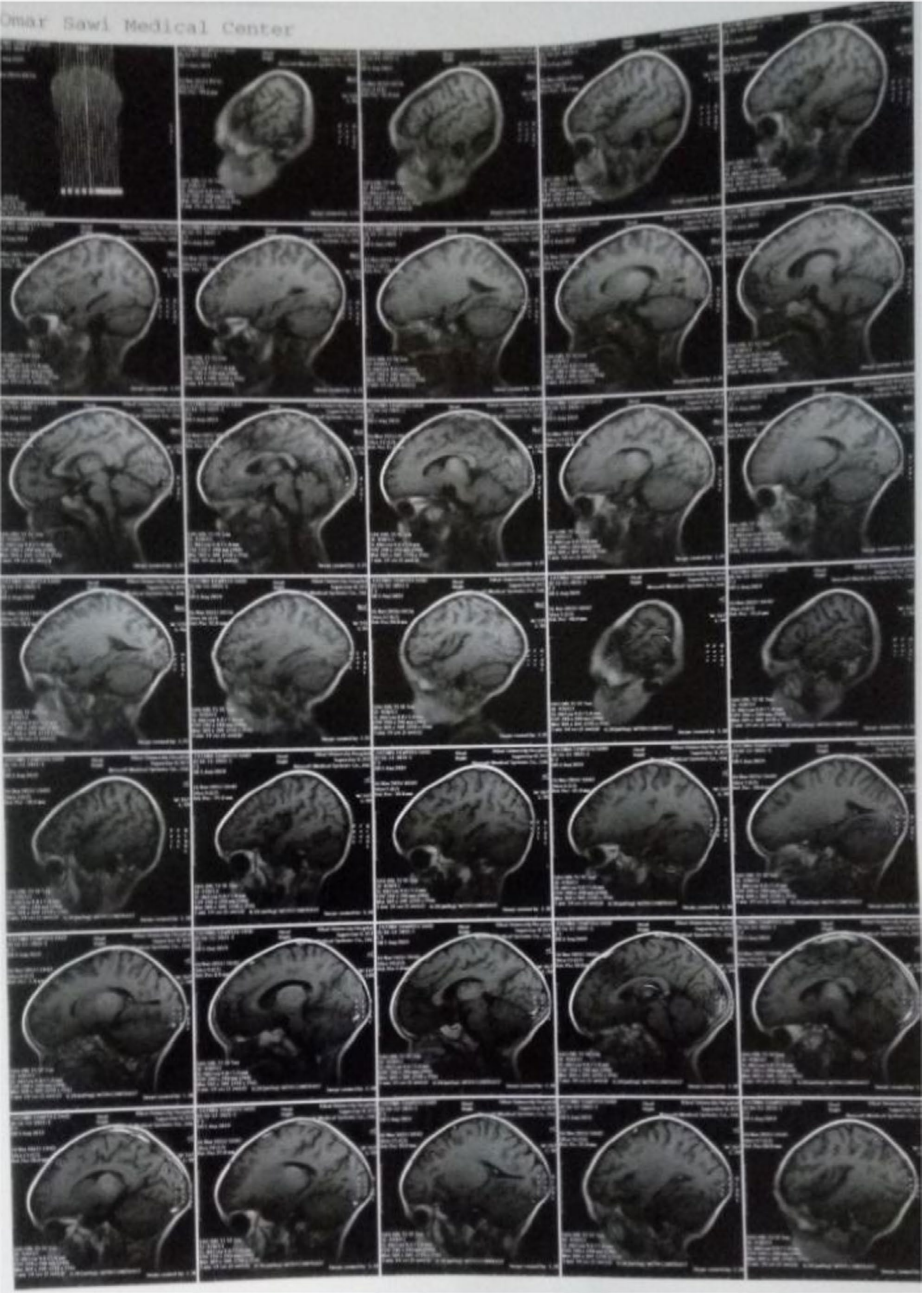
MRI reveals bilateral thalamic lesion due to viral encephalitis or vascular insult

CT of the brain was done which revealed bilateral basal ganglia infarction (Figure 1). MRI was done later which revealed bilateral thalamic lesions due to viral encephalitis or vascular insult (Figure 2, Figure 3).

The patient received acyclovir, meropenem, and ciprofloxacin for 12 days; artesunate for 6 days; and dexamethasone for 9 days, and the patient was put on low‐flow oxygen and regular nasogastric tube feeding. The patient's condition was stable and her conscious level was improving slowly. She was discharged on GCS of 11.

## 3 DISCUSSION

Herein, we report a 2‐year‐old patient with neurological manifestations associated with COVID‐19 infection which is suspected due to the nature of symptoms, the low lymphocyte count, and then confirmed by RT‐PCR. Our findings on CT of the brain revealed bilateral basal ganglia infarction and MRI revealed bilateral thalamic lesions which we assume to be due to direct vascular insult or viral encephalitis. There are very few data about neurological manifestations in pediatric patients with coronavirus disease. Potential differential considerations in a child with acute stroke include stroke, cerebral vasculitis, arterial dissection, genetic disorders, and others.^4^ The exact mechanism of stroke is not known yet. Direct contamination of mucosal linings is the main transmission route for SARS‐CoV‐2. An important role in activating the inflammatory system and chemical release is related to the direct activation of the immune system, platelets, and endothelial cells by SARS‐CoV‐2. Vascular events including stroke may be due to the increased inflammatory responses through various mechanisms.^5^ Vascular hemostasis and blood flow are maintained by endothelial cells. Cells respond to stimuli by secreting pro‐inflammatory cytokines and increasing vascular permeability. Cytokines recruit immune cells and combine with procoagulant factors to formulate a thrombus that captures pathogens, which may contribute to the development of stroke.^5^, ^6^ Concerning encephalitis, there are two main possible routes of entry for SARS‐CoV‐2 to the central nervous system. One is through angiotensin‐converting enzyme 2 receptors located on epithelial cells of the blood–cerebrospinal fluid barrier. The other is retrograde axonal transport of peripheral neural pathways such as through the olfactory mucosa in the nasal cavity.^7^ Encephalitis clinical presentation can mimic stroke clinical presentation as documented in the literature.^8^ Regarding malaria infection, there are not enough data regarding co‐infection, but it is stated in the literature that most patients with co‐infection were symptomatic at presentation. People who live in malaria‐endemic areas and get COVID‐19 infection may be at a more vulnerable statue for developing severe COVID‐19 infection if they were coinfected with malaria,^9^ which we assume attributed to the severity of the inflammatory response hence the patient's presentation. Our patient did not show clinical findings that suggest a multisystem inflammatory syndrome in children (MIS‐C) or a Kawasaki disease‐like presentation, which are common in children with COVID‐19 infection and associated with severe anemia and thrombocytopenia.^10^ We did not find previous evidence in the literature that presents the association between bilateral basal ganglia infarction, COVID‐19, and malaria co‐infection. A case of encephalopathy and bilateral thalamic lesions in a child but with a multisystem inflammatory syndrome associated with COVID‐19 has been reported in the literature.^11^ Although MRI showed bilateral thalamic lesion suggestive for infarction, we did not treat our patient as a patient with stroke; yet, she showed improvement after treating other underlying diseases, which is highly suggestive that the clinical picture of the patient was mainly not due to stroke, but might have been due to the viral encephalitis. Infarction might have been concomitant at the time of presentation and resolved later on its own. Such presentation is considered a challenge for physicians and careful evaluation is required to manage patients properly. We highlight the importance of careful evaluation, especially in areas endemic with malaria, as co‐infection with malaria should be considered. We also emphasize on the importance of a careful neurological evaluation when encountering patients with stroke or stroke‐like presentation. Physicians are to expect extrapulmonary COVID‐19 manifestations and to raise their level of suspicion for the disease.

## 4 CONCLUSION

COVID‐19 infection in the pediatric age group is uncommon, but levels of infection are rising. COVID‐19 infection in pediatrics can present with extrapulmonary manifestations. Encephalitis and stroke can present with a similar clinical picture, so careful evaluation is important for proper management. Malaria co‐infection may contribute to COVID‐19 severity, so immediate action is required for better prognosis.

## CONFLICTS OF INTEREST

All authors declare that there are no conflicts of interest.

## AUTHOR CONTRIBUTIONS

All authors participated in planning the study. YAA and MAA collected data and did investigations and examinations. KAH, MSH, and MMF wrote the first draft. KSE and RSE supervised the process of the study and revised the draft. All authors participated significantly in writing the draft.

## ETHICAL APPROVAL

Ethical approval was obtained from the Sudan State Ministry of Health.

## CONSENT

Considering the child was 2‐year old, both written and verbal consents were obtained from her guardians (parents) in accordance with the general preoperative guidelines as well as consent to be the case of interest in this article.

## Data Availability

The data that support the findings of this study are available with the corresponding author upon reasonable request.
